# The Cut-Off Level of Recombinant Human TSH-Stimulated Thyroglobulin in the Follow-Up of Patients with Differentiated Thyroid Cancer

**DOI:** 10.1371/journal.pone.0133852

**Published:** 2015-07-31

**Authors:** Aldona Kowalska, Iwona Pałyga, Danuta Gąsior-Perczak, Agnieszka Walczyk, Tomasz Trybek, Anna Słuszniak, Ryszard Mężyk, Stanisław Góźdź

**Affiliations:** 1 Endocrinology Department, Holycross Cancer Centre, Kielce, Poland; 2 Laboratory of Tumor Markers, Holycross Cancer Centre, Kielce, Poland; 3 Clinical Oncology Department, Holycross Cancer Centre, Kielce, Poland; Medical University of Gdańsk, POLAND

## Abstract

**Background:**

The treatment of differentiated thyroid cancer (DTC) ends in full recovery in 80% of cases. However, in 20% of cases local recurrences or distant metastases are observed, for this reason DTC patients are under life-long follow-up. The most sensitive marker for recurrence is stimulated thyroglobulin (Tg) which, together with neck ultrasound (US), enables correct diagnosis in nearly all cases of the active disease. For many years the only known stimulation was a 4–5 week withdrawal from the L-T4 therapy (THW). For the last couple of years stimulation with the use of recombinant human TSH (rhTSH) has been available. This method of stimulation may have a significant influence in obtaining the Tg level. However, it is important to determine the cut-off level for rhTSH-stimulated Tg (rhTSH/Tg).

**Materials and Methods:**

This is a retrospective analysis of consecutive patients from one facility who have qualified over a period of two years for repeated radioiodine therapy (RIA). In our facility the ablation effectiveness evaluation is always carried out with the use of rhTSH, with the repeated therapy following THW. Such a procedure enables two Tg measurements in the same patient after both types of stimulation within 4–5 weeks. The obtained values were compared, cut-off levels in THW conditions were used (2.0 ng/ml for patients in remission and 10.0 ng/ml for patients with an active disease). In order to determine the cut-off level for rhTSH/Tg, regression analysis and ROC curves were used.

**Results:**

In 63 patients the Tg measurement of both methods of stimulation were obtained. It was observed that there was a high correlation between rhTSH/Tg and THW/Tg. However, the rhTSH/Tg level was significantly lower than THW/ Tg. The rhTSH/ Tg cut-off levels which corresponded to the 2.0 ng/ml and 10.0 ng/ml limits for THW/Tg were calculated and the values were 0.6 ng/ml and 2.3 ng/ml respectively.

**Conclusions:**

The method of stimulation has a significant impact on the obtained Tg concentrations. The assumed THW/Tg cut off levels must not be transferred to rhTSH/Tg.

## Introduction

Differentiated thyroid cancers (DTC) are being diagnosed increasingly often [1, 2, 3]. The standardized incidence coefficient in Poland according to the National Oncology Registry of the Oncology Centre in Warsaw in 2012 was 8,0 for women and 1.8 for men [4]. The wide-scale introduction of ultrasound diagnostic techniques (US) and cytological screening yields greater detectability of small cancer foci [5], for which reason the increased incidence does not go hand in hand with increased fatality rate [4].

The initial DTC treatment encompasses thyroid surgery and, in selected instances, administration of ^131^I, which results in complete recovery for 80% patients. However, 20% of the patients may have recurrence of the disease, or distant metastases may appear even 40 years after the initial treatment [6]. Such a course of DTC results in life-long follow-up after initial treatment. The frequency and scope of the tests depend on the risk stratification both during the initial treatment and the subsequent stages of the follow-up [7, 8, 9]. The gold standard in DTC diagnosis has for years been the determination of the concentration of stimulated thyroglobulin (Tg), following thyroid hormone treatment withdrawal (THW), as well as whole body scintigraphy (WBS) after administration of the ^131^I [10]. The effect of numerous reports that indicated the limited usefulness of the scintigraphy examination was that many clinicians avoided this tool as a device for monitoring the course of the disease and put the primary focus on the stimulated Tg level [11, 12]. Stimulated Tg is the most sensitive indicator of local recurrence or distant metastases. It has been proved that a false negative result applies only to smaller foci of metastases within regional lymph nodes (LN), which are usually easily detected with US [13]. The only method of Tg stimulation has for many years been a 4–6 week long THW. The following decision algorithms and cut-off thresholds have been developed for this method of stimulation: Tg <1.0–2.0 ng/ml–patient in remission (yearly check-up in the course of LT4), Tg ≥1.0–2.0 ng/ml but <10.0 ng/ml–inconclusive result (^131^I WBS every 1–5 years), Tg ≥10.0 ng/ml–persistent disease (another ^131^I treatment) [14].

Recombinant human TSH (rhTSH)—which enables the stimulation of thyroid cells and neoplastic cells of thyroid origin without the need to THW–was registered in the year 2000. As early as the stage of preliminary tests with the use of rhTSH, it indicated lower Tg concentrations compared to those that were obtained in the same patients after THW [15, 16]. It has not been explicitly defined what level of the rhTSH-stimulated Tg (rhTSH/Tg) need to be assumed as cut-off thresholds while monitoring DTC patients [9, 16, 17, 18, 19, 20].

The purpose of our study was to compare the rhTSH/ Tg level to THW/Tg level in an attempt to estimate what Tg values during the THW are responsible for the concentrations obtained by rhTSH stimulation, as well as an analysis of cut-off thresholds for rhTSH/Tg.

## Materials and Methods

The patients who were included in the tests began therapy at the Holycross Cancer Centre in Kielce during the period between January 2012 and December 2013. The tests were performed on 63 DTC patients (51 women and 12 men) after total thyroidectomy and ^131^I- treatment (RAI) who were qualified for a second RAI after the diagnosis of ablation ineffectiveness. The effectiveness of ablation was evaluated through rhTSH/Tg level, ^131^I whole body scintigraphy (WBS) and US. A visualization of a focal accumulation of ^131^I in the thyroid bed or the concentration of rhTSH/Tg above 2.0 ng/ml (third day after the second injection-day 5) were an indication for repeated therapy. The ^131^I therapy was performed on the patients in the conditions of THW 4–5 weeks after having been qualified according to ESE consensus [7]. All the patients had the TSH and Tg concentration determined again before the administration of the RAI. The obtained mean TSH level was 61,86 μIU/ml median 61,7 μIU/ml at the time of measurement of Tg. The concentration of TSH exceeded 100 μIU/ml in all the patients after administration of rhTSH (day after the second injection-day 3). Based on the THW/Tg level, the patients were divided into 3 groups: Group 1—Tg <2.0 ng/ml, group 2—Tg ≥2.0 ng/ml but <10.0 ng/ml and group 3—Tg ≥10.0 ng/ml. The obtained Tg measurement results were compared with both stimulation types and the rhTSH/Tg levels for patients from the respective groups. The results of the later imaging tests were also scrutinized. The patients with Tg antibodies were excluded from the study.

The study plan was accepted by the Bioethics Committee at the Regional Chamber of Physicians without the necessity to obtain the patients’ formal consent as the data obtained was retrospective data from the patients’ medical history during routine diagnostic procedures while hospitalized. All clinical data were anonymized.

The Tg concentrations were evaluated with the same method of chemiluminescence on the Immulite 2000 xpi Immunoassay System analyzer by Siemens. The method has an analytical sensitivity of 0.2 ng/ml, and functional sensitivity of 0.9 ng/ml.

The basal serum specimen for each patient was screened for the presence of Tg antibodies using a chemiluminescence method on the Immulite 2000 xpi Immunoassay System analyzer by Siemens. Analytical sensitivity: 2.2 IU/ml. Neck ultrasonography was performed with the use of devices featuring a color doppler function: Siemens Versa pro and Hitachi EUB-6500, with a high frequency linear probe (7.5 MHz). The WBS was performed with a Symbia T2 gamma camera by Siemens with the use of a high energy collimator with a scanning speed of 10 cm/min. The diagnostic WBS was performed 72 hours after the administration of 180MBq ^131^I, and the post-therapy WBS on day 5 after the RAI

### Statistical Analysis

Basic statistics were defined for the analyzed parameters (mean, SD, median, quartiles, percentiles, range). Spearman's rank correlation, regression analysis, the Receiver Operating Characteristic (ROC) analysis and the Wilcoxon test were used for analyzing cut-off point’s sensitivity, specificity, positive predictive value (PPV), negative predictive value (NPV) and its 95% confidence intervals (CI) were calculated.The software used for calculations was MedCalc Statistical Software version 15.2.1 (MedCalc Software bvba, Ostend, Belgium; http://www.medcalc.org; 2015)

## Results

Having assumed the aforementioned cut-off levels for the THW/Tg, the number of examined patients was 42 (66.7%) in group 1; 8 (12.7%) in group 2, and 13 (20.6%) in group 3. The rhTSH/Tg and THW/Tg concentrations in respective groups are summarized in Table 1.

**Table 1:** Recombinant human thyrotropin-stimulated thyroglobulin (rhTSH/Tg) levels in groups of patients divided in accordance to thyroid hormone withdrawal-stimulated thyroglobulin (THW/Tg) cut-off levels.

THW/Tg (ng/ml) cut-off level	N	Parameter	THW/Tg (ng/ml)	rhTSH/Tg (ng/ml)	p-value
< 2	42	Mean (SD) Median	0,2 (0,4) 0,0	0,1 (0,3) 0,0	0,0215
≥2 and <10	8	Mean (SD) Median	4,50 (2,3) 4,4	1,6 (1,1) 1,2	0,0078
≥10	13	Mean (SD) Median	35,4 (12,5) 32,3	7,6 (5,6) 6,6	0,0002

N- number of patients

**Fig 1:**
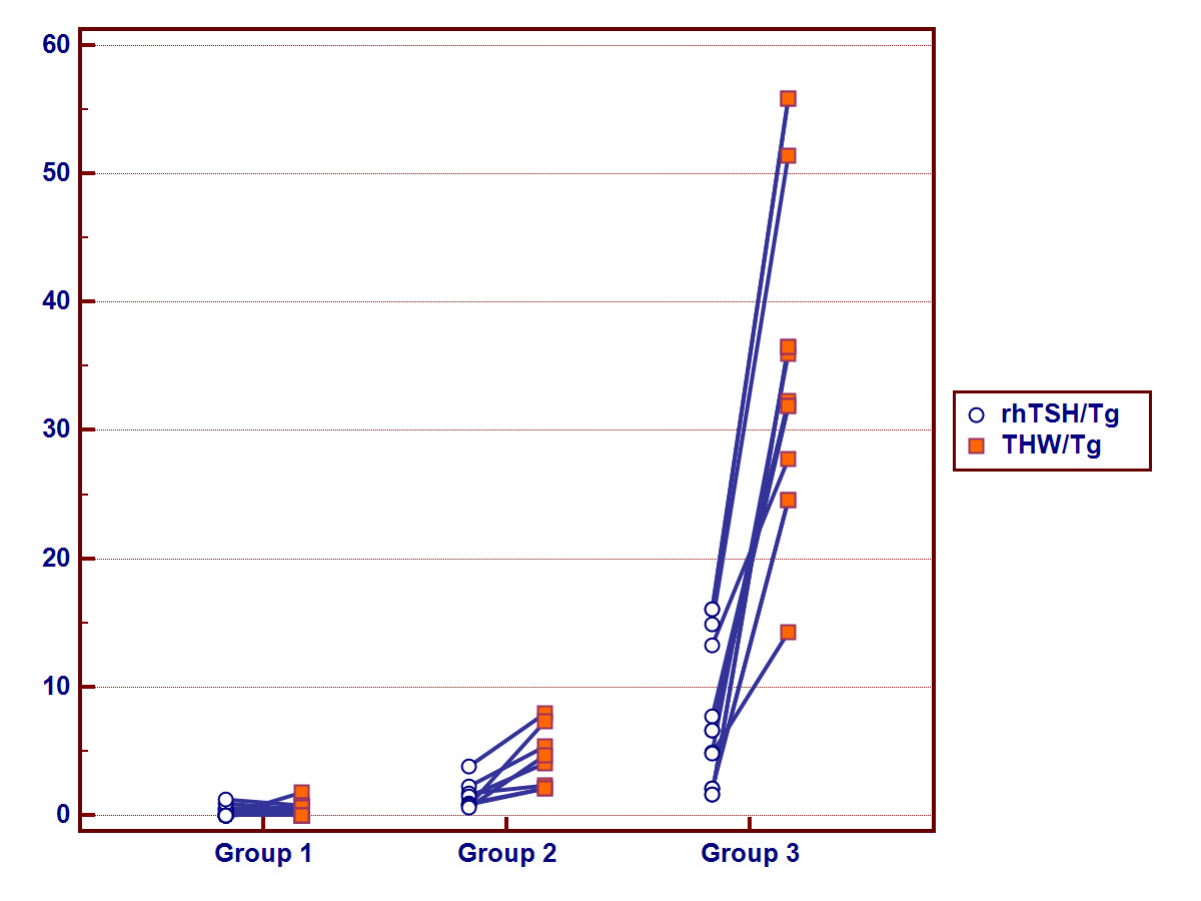
Recombinant human thyrotropin-stimulated thyroglobulin (rhTSH/Tg) and thyroid hormone withdrawal-stimulated thyroglobulin (THW/Tg) in the same patients in three groups: Group 1- THW/Tg < 2ng/ml; Group 2-THW/Tg ≥ 2ng/ml and < 10ng/ml; Group 3- THW/Tg ≥ 10 ng/ml.

The method of stimulation had a significant impact on Tg concentration (see Table 1). Higher levels of Tg were obtained through THW stimulation than with rhTSH stimulation. The higher the Tg level, the higher the Tg difference concentration related to the method of stimulation (Fig 1).

Further analysis of the image results are summarized in Table 2.

**Table 2:** Results of imaging studies in involved groups of patients.

Groups involved	NS	US	WBS	CT	PET	Biochemically persistent disease rhTSH/Tg>10
Group 1 (42)	42 (+)	42 (-)	(-)	ND	ND	0
Group 2 (8)	8 (+)	8(-)	1 (+) Th5 7(-)	4(-) 3 ND	1 (-) 7 ND	0
Group 3 (13)	2 (+) 11 (-)	13(-)	8(+): 3 lung, 5 M; 5(-)	2(+): 2 lung; 11(-)	3(+): 2 M LN, 1 neck LN; 2(-); 8 ND	2: 14,9 ng/ml 13,3 ng/ml

NS- neck scan, US- ultrasound, WBS- ^131^I—whole body scan, CT- computed tomography, PET- positron emission tomography with ^18^ F-FDG, rhTSH/Tg–recombinant human thyrotropin-stimulated thyroglobulin, ND- not done, M- mediastinum, LN- lymph node

None of the group 1 patients had uptake outside the thyroid bed on the diagnostic WBS (ineffectiveness of the first ablation). In group 2, apart from the radioiodine accumulation in the thyroid bed, only one female patient (pT4N1) had a focal accumulation of ^131^I in Th5 visualized in the ^131^I-SPECT examination which were not confirmed in other image tests (rhTSH/ Tg and THW/ Tg respectively 3.83–7.93 ng/ml).

In 11 patients from group 3 (85%) distant or regional metastases were observed (3 lungs visible in WBS: 2 confirmed by CT scan; 7 mediastinum: 5 patients visible only in WBS, 2 only in PET; 1 lateral cervical LN in PET). In 2 patients from this group no recurrence or distant metastases were diagnosed (rhTSH/Tg and THW/Tg respectively: 14.9–51.4 ng/ml and 13.3–27.8 ng/ml—biochemically persistent disease).

A high correlation (r = 0.902; p<0.0001) between THW/Tg and rhTSH/Tg has been reported.

**Fig 2:**
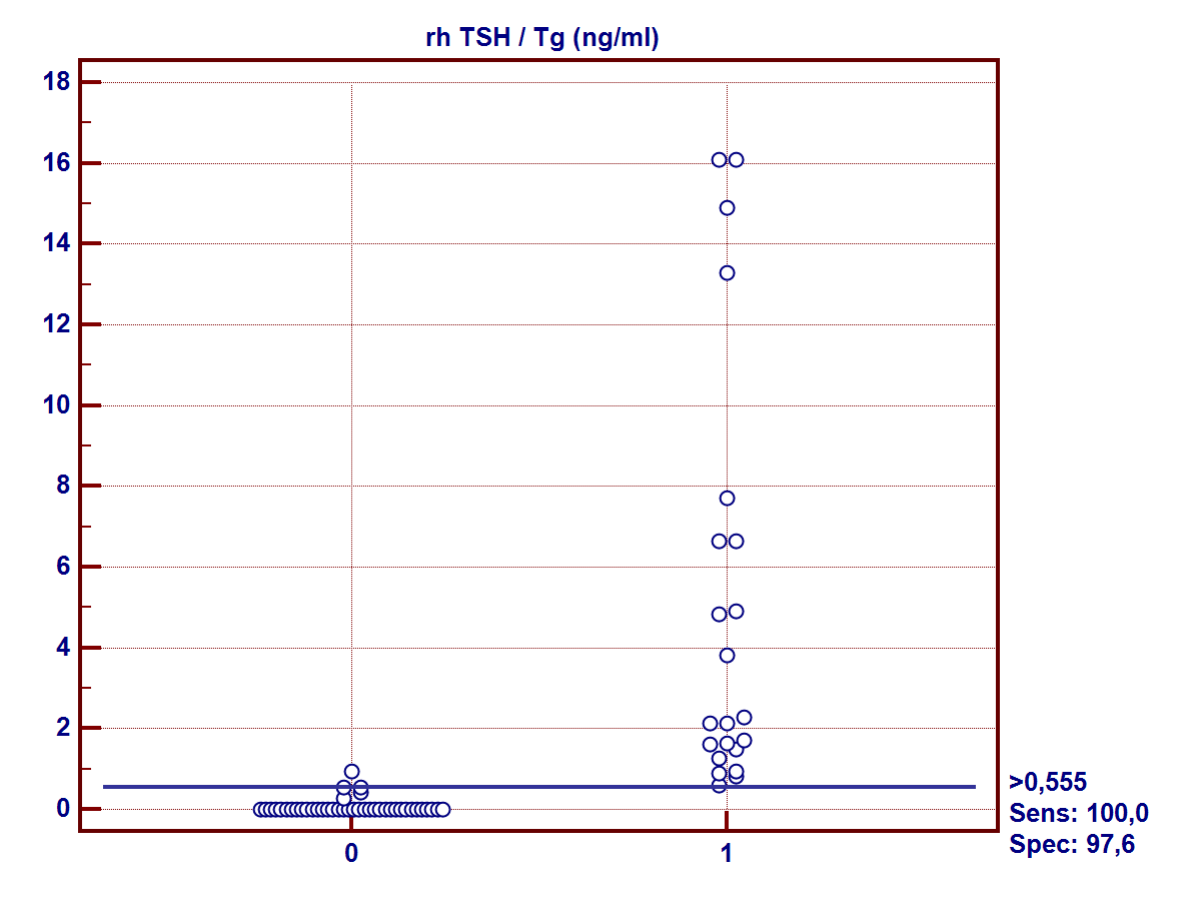
Recombinant human thyrotropin-stimulated thyroglobulin (rhTSH/Tg) sensitivity and specificity for cut-off level > 0.6 ng/ml. 0-Group of patients with thyroid hormone withdrawal-stimulated thyroglobulin (THW/Tg) < 2 ng/ml 1-Group of patients with thyroid hormone withdrawal-stimulated thyroglobulin (THW/Tg) ≥ 2 ng/ml

**Fig 3:**
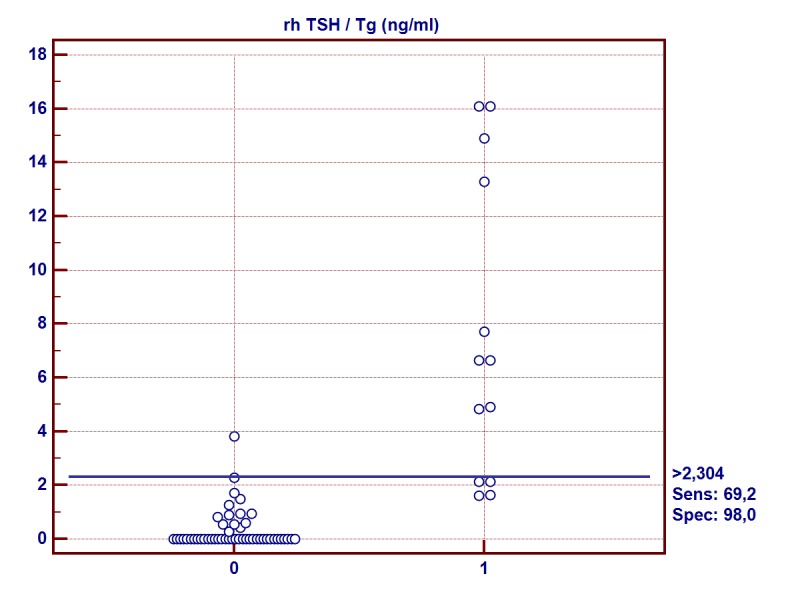
Recombinant human thyrotropin-stimulated thyroglobulin (rhTSH/Tg) sensitivity and specificity for cut-off level > 2.3 ng/ml. 2-Group of patients with thyroid hormone withdrawal-stimulated thyroglobulin (THW/Tg) < 10 ng/ml 3-Group of patients with thyroid hormone withdrawal-stimulated thyroglobulin (THW/Tg) ≥ 10 ng/ml

Based on the regression equation (y = 0,1413+0,2223*x) the TSH/Tg (y) values for THW/Tg (x) values were determined in the group involved: 2.0 ng/ml and 10.0 ng/ml, which corresponded to 0.6 ng/ml and 2.3 ng/ml respectively (Figs 2 and 3).

As a result of the ROC analysis, cut-off levels for rhTSH/Tg were determined for which the sensitivity and specificity showed the highest values. The results are summarized in Table 3.

**Table 3:** Thyroid hormone withdrawal-stimulated thyroglobulin (THW/Tg) cut-off levels and corresponding recombinant human thyrotropin-stimulated thyroglobulin (rhTSH/Tg) cut-off levels in regression analysis and ROC analysis.

THW/Tg (ng/ml)	rhTSH /Tg ng/ml1 (95% CI)	Criterion values rhTSH/Tg ng/ml	Sensitivity (95% CI)	Specificity (95% CI)	PPV (95% CI)	NPV (95% CI)
2	0,586 [~0,6]	>0,555	100	98	96	100
	(-0,021–0,650)	[~0,6]	(84–100)	(87–100)	(35–77)	(91–100)
10	2,364 [~2,4]	>2,27	69	98	90	92
	(1,503–2,683)	[~2,3]	(39–91)	(89–100)	(56–100)	(82–98)

rhTSH/Tg cut-off level determined based on regression equation y = 0,1413+0,2223*x where: y = rhTSH/Tg; x = THW/Tg

PPV—Positive predictive value; NPV—Negative predictive value

## Discussion

In recent decades a marked increase of DTC incidence has been observed [1, 2, 3]. The prognosis for DTC is positive, but more than 20% of the patients experience a local recurrence or distant metastases, sometimes after many years. These observations have their origin in large cohort studies from the second half of the 20th century and are a basis for life-long follow-up recommendations for all DTC patients [6]. The DTC patient structure has changed over recent years. The widespread use of US and fine-needle aspiration cytology (FNAC) techniques has brought about a marked improvement in the diagnosis of DTC at an early clinical stage [5]. The determination of the THW/Tg level and WBS has become a gold standard in monitoring the course of DTC [10]. Our observations have confirmed the high clinical relevance of the THW/Tg cut-off levels (2.0 ng/ml and 10.0 ng/ml) and indicated that there is a necessity to determine what rhTSH/Tg level corresponding to THW/Tg cut-offs.

Taking into account hypothyroidism and ^131^I related burden for the patients, it is essential to adjust the method of oncological monitoring to the risk of relapse or metastases. For most low-risk patients, remission is confirmed by just evaluating the rhTSH/Tg and US of the neck [9, 21]. This monitoring method allows patients to avoid hypothyroidism and exposure to ionizing radiation. Assigning rhTSH/Tg—the main indicator for remission or recurrence, requires particular diligence in the use of this marker. While evaluating the Tg concentration it needs to be remembered that the method of stimulation has a significant impact on the values obtained. Our observations corroborate earlier reports by Pacini et al. [15] and Haugen et al. [16] and indicate that rhTSH/Tg levels are markedly lower than THW/Tg levels. Increased or decreased Tg concentrations over time are a significant feature of clinical analysis [7, 8]. If the Tg concentration is identifiable, then varied methods of stimulation must not be used in order to evaluate the concentrations of this marker over time. Our results demonstrated more significant differences for higher Tg concentrations. However, we have observed a high concordance between both stimulation methods in patients with a Tg concentration below the threshold for the analytical sensitivity of the method. For 36 such patients, the THW stimulation never caused any increase in Tg concentration in 29 patients, an increase to the maximum value of 0.6 ng/ml in 6 patients, or the maximum value of 1.8 in 1 patient. Different authors adopt different cut-off levels for rhTSH/Tg for the evaluation of ablation effectiveness, from 0.8 ng/ml by Taïeb [17], through 1.0 ng/ml by Schlumberger and Chianella [18, 22] to 2.0 ng/ml in Mallick and Paccini [19, 7].

The more frequent use of rhTSH in clinical practice raises the issue of cut-off levels for rhTSH/Tg for a persistent disease or relapse. Numerous clinical observations with the THW/Tg evaluation have enabled the determination of the >10 ng/ml value as a cut-off level for a persistent disease. This value correlated with the clinical condition very well. However, it may not be treated as valid with rhTSH stimulation. The recommended cut-off level for a persistent disease in examinations is >5.0 ng/ml [21]. Such a value would not correctly qualify 6 out of 13 patients from the patient group with a persistent disease (rhTSH/Tg 1.6–4.9). A higher value > 10 ng/ml is indicated by Momesso [9], which would cause incorrect qualification of up to 9 out of 13 patients (rhTSH/Tg 1.6 ng/ml—7.71 ng/ml). Our results come closest to those of Hugen's [16], where it is indicated that rhTSH/Tg- 2.0 ng/ml is a value that indicates the presence of persistent disease or remnant thyroid tissue. The study by Cláudio et al. revealed that in 60% of the patients with rhTSH/Tg concentration >2.0 ng/ml metastases was diagnosed [23]. Adopting the 2.0 ng/ml cut-off rhTSH/Tg level would enable correct qualification of 12 out of 13 patients with a persistent disease. David A et al. demonstrated that every rhTSH/Tg concentration increase over 1.0 ng/ml may indicate a relapse or a persistent disease. The increase in the rhTSH/Tg level to 1.0–5.0 ng/ml in the study group analyzed by the authors was linked to the presence of metastases in 30% of the patients and an increase in the value to over 5.0 ng/ml up to 81% [24]. The increase of the rhTSH/Tg level to over 1.0 ng/ml in our material was observed in all patients with the diagnosed persistent disease and in 4 out of 49 patients with ineffective ablation without any other disease foci in the image tests.

Our analysis was an attempt to determine the cut-off levels for rhTSH/Tg and was based on comparison with the clinically proven thresholds for THW/Tg. It indicates that the rhTSH/Tg value with the highest sensitivity and specificity that corresponds to the value 2.0 ng/ml for THW/Tg is 0.6 ng/ml, whereas for the value of 10.0 ng/ml it is 2.3 ng/ml.

The relatively narrow group of patients is a limitation of our study. However, all patients who had Tg determinations after both types of stimulation within 4–5 weeks were put under observation. In our facility, we evaluate the effectiveness of ablation with the use of rhTSH, whereas the therapy is conducted in THW conditions due to limited access to rhTSH. Another possible setback is an inaccurate Tg concentration measurement for a value lower than the functional sensitivity of the test. For the method used in our laboratory, the functional sensitivity amounted to 0.9 ng/ml. The evaluations conducted of the accuracy of Tg level (repeatability and reproducibility) for the value of 0.4 ng/ml revealed a coefficient of variation of 32%. The solution to this problem may be the use of ultrasensitive tests for Tg determinations.

## Conclusions

The method of Tg stimulation had a significant impact on the Tg values obtained. The rhTSH/Tg were markedly lower than those obtained with THW stimulation. On account of major discrepancies, the same cut-off thresholds should not be used for different stimulation methods. We recommend the rhTSH/Tg cut-off level for those patients who remain free of disease to be ≤ 0.6 ng/ml, whereas for those patients with a persistent disease ≥2.3 ng/ml. It is essential to perform tests in a larger group of patients to corroborate the correctness of the values that we have adopted.
